# Pre- and/or Intra-Operative Prescription of Diuretics, but Not Renin-Angiotensin-System Inhibitors, Is Significantly Associated with Acute Kidney Injury after Non-Cardiac Surgery: A Retrospective Cohort Study

**DOI:** 10.1371/journal.pone.0132507

**Published:** 2015-07-06

**Authors:** Miho Tagawa, Ai Ogata, Takayuki Hamano

**Affiliations:** 1 Department of Nephrology, Kyoto Katsura Hospital, 17 Yamada-hirao-cho, Nishikyo-ku, Kyoto, 6158256, Japan; 2 First Department of Internal Medicine, Nara Medical University, 840 Shijo-cho, Kashihara-shi, Nara, 634–8522, Japan; 3 Department of Nephrology, Kyoto City Hospital, 1–2, Higashitakada-cho, Mibu, Nakagyo-ku, 6048845, Japan; 4 Department of Comprehensive Kidney Disease Research, Osaka University Graduate School of Medicine, 2–2, Yamadaoka, Suita-shi, Osaka, 5650871, Japan; School of Public Health of University of São Paulo, BRAZIL

## Abstract

**Background and Objectives:**

Pre- and/or intra-operative use of diuretics, angiotensin-converting enzyme inhibitors (ACE-I) or angiotensin II receptor blockers (ARB) constitutes a potentially modifiable risk factor for postoperative acute kidney injury (AKI). It has been studied whether use of these drugs predicts AKI after cardiac surgery. The objective of this study was to examine whether administration of these agents was independently associated with AKI after non-cardiac surgery.

**Design, Setting, Participants, and Measurements:**

This was a retrospective observational study. Inclusion criteria were adult patients (age ≥ 18) who underwent non-cardiac surgery under general anesthesia from 2007 to 2009 at Kyoto Katsura Hospital. Exclusion criteria were urological surgery, missing creatinine values, and preoperative dialysis. The exposures of interest were pre- and/or intra-operative use of diuretics or ACE-I/ARB. Outcome variables were postoperative AKI as defined by the AKI Network (increase in creatinine ≥ 0.3 mg/dL or 150% within 48 hours, or urine output < 0.5 ml/kg/hour for > 6 hours). Multivariable logistic regression analyses were conducted and adjusted for potential confounders. Propensity scores (PS) for receiving diuretics or ACE-I/ARB therapy were estimated and PS adjustment, PS matching, and inverse probability weighting were performed.

**Results:**

There were 137 AKI cases (5.0%) among 2,725 subjects. After statistical adjustment for patient and surgical characteristics, odds (95% CI) of postoperative AKI were 2.07 (1.10-3.89) (p = 0.02) and 0.89 (0.56-1.42) (p = 0.63) in users of diuretics and ACE-I/ARB, respectively, compared with non-users. PS adjustment, PS matching, and inverse probability weighting yielded similar results. The effect size of diuretics was significantly greater in the patients with lower propensity for diuretic use (p for interaction < 0.1).

**Conclusions:**

Prescription of diuretics, but not ACE-I/ARB, was independently associated with postoperative AKI after non-cardiac surgery, especially in patients with low propensity for diuretic use. It might be reasonable to withhold preoperative diuretics in these patients.

## Introduction

Postoperative acute kidney injury (AKI) is a serious complication of surgical procedures that is associated not only with short-term increases in mortality [1–3] but also with long-term complications such as development of chronic kidney disease [4]. Predictors of postoperative AKI have been extensively studied in cardiac surgery [5–22], and to a lesser extent in non-cardiac surgery [23–30].

As reported in previous studies, these predictors include age, female sex, types of surgery (valvular surgery versus coronary artery bypass grafting in cardiac surgery and intra-thoracic or intra-abdominal surgery versus others in non-cardiac surgery), emergency surgery, preoperative renal dysfunction, body mass index (BMI), smoking, diabetes mellitus (DM), the use of insulin, hypertension, chronic obstructive pulmonary disease (COPD), atrial fibrillation, peripheral arterial disease (PAD), cerebrovascular disease (CVA), coronary artery disease (CAD), preoperative hematocrit, coagulopathy, thrombocytopenia, the use of vasopressors, left ventricular dysfunction, pre- and/or intra-operative use of iodinated contrast, diuretics, angiotensin converting enzyme inhibitors (ACE-I) and angiotensin receptor blockers (ARB) [5–30]. Among these predictors of AKI, pre- and/or intra-operative use of diuretics, ACE-I or ARB is possibly modifiable. There are several studies that examined the association of ACE-I or ARB with AKI after cardiac surgery with conflicting results [17–19, 22], while a meta-analysis showed that the use of ACE-I/ARB was significantly associated with increased odds of postoperative AKI and mortality [21]. The studies that examined the association between preoperative use of ACE-I/ARB and postoperative AKI in non-cardiac surgery were of small sample size [26, 28]. To our knowledge, no studies have investigated whether use of diuretics is associated with postoperative AKI in non-cardiac surgery. We hypothesized that pre- and/or intra-operative use of diuretics or ACE-I/ARB is independently associated with AKI after non-cardiac surgery, and tested this hypothesis in a single-center, retrospective cohort study.

## Materials and Methods

### Study Design, Settings and Patients

This was a single center, retrospective cohort study. Inclusion criteria were adult patients (age ≥ 18) who underwent non-cardiac surgery under general anesthesia from 2007–2009 at Kyoto Katsura Hospital. Patients were excluded if they had undergone urological surgery (because changes in creatinine due to nephrectomy or ureteral manipulation are likely to be caused by different mechanisms from those underlying other postoperative AKI), were missing creatinine values within 1 week preoperatively or 48 hours postoperatively, or had undergone dialysis preoperatively. The exposures of interest were pre- and/or intra-operative use of diuretics, ACE-I and/or ARB. Intra-operative use of diuretics was included as it is a common practice in Japan to administer diuretics intra-operatively to maintain urine output. If urine output increases in response to diuretic administration, and serum creatinine does not increase postoperatively, the patient is not diagnosed with postoperative AKI. Thus, intra-operative use of diuretics is not a result of AKI but considered to be a risk factor for postoperative AKI. The outcome variable was postoperative AKI as defined by the AKI Network (increase in creatinine ≥ 0.3 mg/dL or 150% within 48 hours, or urine output < 0.5 ml/kg/hour for > 6 hours) within 2 days postoperatively [31]. The data was collected from review of medical charts.

### Definitions

Pre- and/or intra-operative use of diuretics and ACE-I/ARB was defined as the use of these agents from the preoperative period through the end of surgery, as confirmed by medication lists in medical charts. Operations were divided into 4 categories: intra-thoracic surgery, intra-abdominal surgery, surgery with large fluid shift and others. Surgery with large fluid shift included total hysterectomy and replacement of major joints such as hips and knees. Estimated glomerular filtration rate (eGFR) was calculated using the equation developed for Japanese populations by the Japanese Society of Nephrology [32], based on the preoperative creatinine value closest to the time of surgery. The use of vasopressors (norepinephrine, epinephrine, phenylephrine, dopamine and ephedrine) was defined as intra-operative administration of these agents. The use of non-steroidal anti-inflammatory drugs (NSAIDs) and iodinated contrast was defined as the use of these agents within 48 hours pre-operatively or their administration intra-operatively. Chronic kidney disease (CKD) was defined as eGFR < 60 ml/min/1.73m^2^.

### Statistical Methods

Continuous variables were expressed as median with interquartile range and were compared using the Mann-Whitney test. Categorical variables were expressed as number with percentage and were compared using the Chi-square test. A multivariable logistic regression model was used to estimate odds ratios and 95% confidence intervals (CIs) of postoperative AKI following the use of diuretics or ACE-I/ARB, with non-users of these agents as a reference. Pre-specified covariates forced into the models included pre- and/or intra-operative use of diuretics and ACE-I/ARB. Other covariates were selected using the backward elimination method by likelihood ratio test. CKD was used as a covariate rather than eGFR as the latter was not significantly associated with postoperative AKI when analyzed as a continuous variable. Left ventricular ejection fractions were divided into 3 categories (> 40%, ≤ 40%, or missing), as echocardiograms were not performed for patients with low cardiac risk and thus missing echocardiogram was not random. Model fit was assessed with the Hosmer-Lemeshow goodness-of-fit test. As there were only 137 patients with the outcome (postoperative AKI), it was not possible to include several possible confounders in the logistic regression analyses. To maximize the inclusion of possible confounders, the analyses were also performed using propensity score (PS). PS for diuretic and ACE-I/ARB use were derived from all the variables in Table 1. The use of ACE-I/ARB or diuretics was included in the model when generating PS for diuretics and ACE-I/ARB, respectively. The discrimination of the model was assessed using the c-statistic and receiver operating characteristic curve. We performed three different sensitivity analyses: PS adjustment, PS matching, and inverse probability weighting. For PS adjustment, PS quintiles were used rather than the logit of the PS as a continuous variable, because the association between PS and odds of postoperative AKI proved to be nonlinear (S1 Fig). The PS quintiles were treated as a categorical variable. PS matching was also performed. The users of ACE-I/ARB and diuretics were matched with non-users on the logit of PS (+/- 0.2 SD) (user: non-user = 1:2) by a greedy matching method. The odds of postoperative AKI in a propensity-matched cohort were examined using logistic regression analyses. Interaction terms were added to multivariable logistic regression analyses to determine whether the presence of CKD was an effect modifier of the association between diuretics, ACE-I/ARB use and AKI, and whether the use of ACE-I/ARB or the propensity for diuretic use was an effect modifier of the association between diuretic use and AKI. Values of p ≤ 0.05 were considered statistically significant except for interactions where p ≤ 0.10 was considered statistically significant [33, 34].

**Table 1:** Clinical characteristics of patients.

	No AKI (n = 2,588)	AKI (n = 137)	p
Age	63 (52–72)	71 (61–76)	<0.001
Male sex	1281 (49.5)	92 (67.2)	<0.001
Intra-thoracic surgery Intra-abdominal surgery Surgery with large fluid shift Others	478 (18.5) 1140 (44.0) 441 (17.0) 529 (20.5)	36 (26.3) 79 (57.7) 16 (11.7) 6 (4.3)	<0.001
Emergency surgery	169 (6.5)	23 (16.8)	<0.001
eGFR (ml/min/1.73m^2^)	77.7 (66.3–93.6)	67.9 (53.7–85.5)	<0.001
eGFR ≥ 60 30 ≤ eGFR < 60 15 ≤ eGFR < 30 eGFR < 15	2241 (86.6) 335 (12.9) 10 (0.4) 2 (0.1)	88 (64.2) 42 (30.7) 6 (4.4) 1 (0.7)	
Body mass index	22.0 (19.8–24.7)	23.3 (21.0–25.4)	0.003
Smoking	652 (25.2)	28 (20.4)	0.21
Diabetes Mellitus	392 (15.1)	42 (30.7)	<0.001
Insulin	48 (1.9)	11 (8.0)	<0.001
Hypertension	1030 (39.8)	87 (63.5)	<0.001
COPD	203 (7.8)	18 (13.1)	0.027
Atrial fibrillation	67 (2.6)	9 (6.6)	0.006
Peripheral arterial disease	32 (1.2)	1 (0.7)	0.60
Cerebrovascular disease	91 (3.5)	19 (13.9)	<0.001
Coronary artery disease	154 (6.0)	16 (11.7)	0.007
Hematocrit (%)	39.2 (35.8–42.2)	37.7 (33.0–41.2)	0.001
INR > 1.5	14 (0.5)	2 (1.5)	0.17
Platelet < 150,000/μl	241 (9.3)	24 (17.5)	0.002
Vasopressors	1273 (49.2)	97 (70.8)	<0.001
Left ventricular ejection fraction >40% ≤40% missing	1298 (50.2) 7 (0.3) 1283 (49.5)	77 (56.2) 0 (0) 60 (43.8)	0.33
NSAIDs	2318 (89.6)	116 (84.7)	0.071
Contrast	160 (6.2)	16 (11.7)	0.011
Diuretics	83 (3.2)	16 (11.7)	<0.001
ACE-I/ARB	420 (16.2)	40 (29.2)	<0.001

Data are shown as median (interquartile range) or number (%). P values were determined using the Mann-Whitney U test or Chi-square test. AKI: acute kidney injury, eGFR: estimated glomerular filtration rate, COPD: chronic obstructive pulmonary disease, INR: international normalized ratio of prothrombin time, NSAIDs: non-steroidal anti-inflammatory drugs, ACE-I: angiotensin-converting enzyme inhibitor, ARB: angiotensin receptor blocker

All analyses were performed using SPSS version 19.0 (SPSS Inc, Chicago, IL).

### Ethics Statement

The study protocol and waiver of consents were approved by the Ethics Committee of Kyoto Katsura Hospital and the study was conducted in accordance with the Declaration of Helsinki.

## Results

During the study period, 3,455 patients underwent non-cardiac surgeries under general anesthesia at Kyoto Katsura Hospital. Of these, the following 730 patients were excluded: 196 who underwent urological surgeries, 504 without available creatinine values within 1 week preoperatively and/or 48 hours postoperatively, 20 who had undergone dialysis preoperatively, and 10 who had incomplete data. Thus 2,725 patients were eligible for analyses.

One hundred thirty-seven patients (5.0%) developed AKI and 3 (0.1%) required renal replacement therapy. In-hospital mortality was 1.2% (33/2725) [0.8% (21/2588) for patients without AKI and 8.0% (11/137) for patients with AKI]. Patients’ clinical characteristics are shown in Table 1. Preoepratively, 396 patients (14.5%) had CKD stage 3 or more. Patients with AKI were significantly older, more likely to be males, more likely to have undergone intra-thoracic, intra-abdominal or emergency surgeries, and more likely to have significantly lower eGFR, higher BMI and more comobidities, preoperatively. Significantly more patients with AKI received vasopressors intraoperatively, iodinated contrast, diuretics or ACE-I/ARB pre- and/or intra-operatively. No patients skipped diuretics or ACE-I/ARB on the day of surgery. Twenty-two patients were given diuretics during surgery to maintain urine output. No patients received ACE-I/ARB only on the day of surgery.

Multivariable logistic regression analyses were performed using pre- and/or intra-operative use of diuretics and ACE-I/ARB as well as covariates selected by backward elimination using the likelihood ratio test. The p value by Hosmer-Lemeshow test was 0.51. Pre- and/or intra-operative use of diuretics, but not the use of ACE-I/ARB, was significantly associated with development of AKI (Table 2). Other covariates significantly associated with the development of AKI included male sex, intra-thoracic surgery, intra-abdominal surgery, surgery with large fluid shifts, emergency surgery, the presence of CKD, BMI, the use of insulin, hypertension, CVA, pre-operative hematocrit and intra-operative use of vasopressors. The association of pre- and/or intra-operative use of diuretics or ACE-I/ARB and postoperative AKI was not significantly modified by the presence of CKD stage 3 or more (eGFR < 60 ml/min/1.73 m^2^) (p for interaction [diuretic use * CKD] = 0.25 and p for interaction [ACE/ARB use * CKD] = 0.51).

**Table 2:** Multivariable logistic regression analysis.

	Odds ratio (95% CI)	p
Diuretics	2.07 (1.10–3.89)	0.02
ACE-I/ARB	0.89 (0.56–1.42)	0.63
Age	1.02 (1.00–1.03)	0.10
Male sex	1.92 (1.26–2.92)	0.002
Intra-thoracic surgery Intra-abdominal surgery Surgery with large fluid shift Others	8.16 (3.32–20.10) 3.61 (1.52–8.56) 3.66 (1.37–9.79) 1 (reference)	<0.001 0.004 0.01
Chronic kidney disease*	1.93 (1.28–2.93)	0.002
Emergency surgery	2.57 (1.49–4.43)	0.001
Body mass index	1.08 (1.03–1.14)	0.003
Insulin	3.40 (1.57–7.36)	0.002
Hypertension	1.84 (1.18–2.86)	0.007
Cerebrovascular disease	2.25 (1.25–4.02)	0.006
Hematocrit (%)	0.96 (0.92–0.99)	0.02
Vasopressors	1.82 (1.21–2.73)	0.004

ACE-I: angiotensin-converting enzyme inhibitor, ARB: angiotensin receptor blocker

*Chronic kidney disease was defined by estimated glomerular filtration rate < 60 ml/min/1.73m^2^.

PS for the use of diuretics and ACE-I/ARB were derived from all the variables in Table 1. C-statistics for diuretics and ACE-I/ARB were 0.86 and 0.75, respectively. Multivariable logistic regression analyses using PS quintiles and the use of diuretics and ACE-I/ARB showed that pre- and/or intra-operative use of diuretics, but not ACE-I/ARB, was significantly associated with the development of postoperative AKI (Table 3). P values by the Hosmer-Lemeshow test for diuretics and ACE-I/ARB were 0.95 and 0.86, respectively.

**Table 3:** Comparison of odds ratio of postoperative acute kidney injury estimated by different statistical analyses.

		Odds ratio (95% CI)	p
Diuretics	Multivariable logistic regression	2.07 (1.10–3.89)	0.02
	Adjustment for PS quintiles	2.35 (1.30–4.24)	0.005
	PS matching	2.36 (1.06–5.24)	0.04
	Inverse probability weighting	2.97 (1.29–6.80)	0.01
ACE-I/ARB	Multivariable logistic regression	0.89 (0.56–1.42)	0.63
	Adjustment for PS quintiles	0.98 (0.63–1.53)	0.92
	PS matching	0.75 (0.43–1.32)	0.32
	Inverse probability weighting	1.25 (0.66–2.34)	0.50

Propensity scores for diuretics and ACE-I/ARB were derived using all the variables in Table 1. PS: propensity score, ACE-I; angiotensin-converting enzyme inhibitor, ARB: angiotensin receptor blocker

PS matching yielded pairs of 94 diuretic users and 188 non-users, and 309 users of ACE-I/ARB and 618 non-users. Demographics of the propensity-matched patients were well-balanced (S1 and S2 Tables). In the propensity-matched cohort, pre-operative use of diuretics, but not the use of ACE-I/ARB, was significantly associated with post-operative AKI (Table 3). We failed to match about one-third of the patients receiving ACE-I/ARB because of a considerable difference in the distribution of PS between the users and non-users. Thus, inverse probability weighting was also performed as a sensitivity analysis. The results were similar to those of other analyses (Table 3). Excluding patients whose inverse-probability weight was < 1 percentile and > 99 percentile or excluding patients whose PS do not overlap among users and non-users of ACE-I/ARB or diuretics did not significantly change the results (S3 Table).

**Fig 1:**
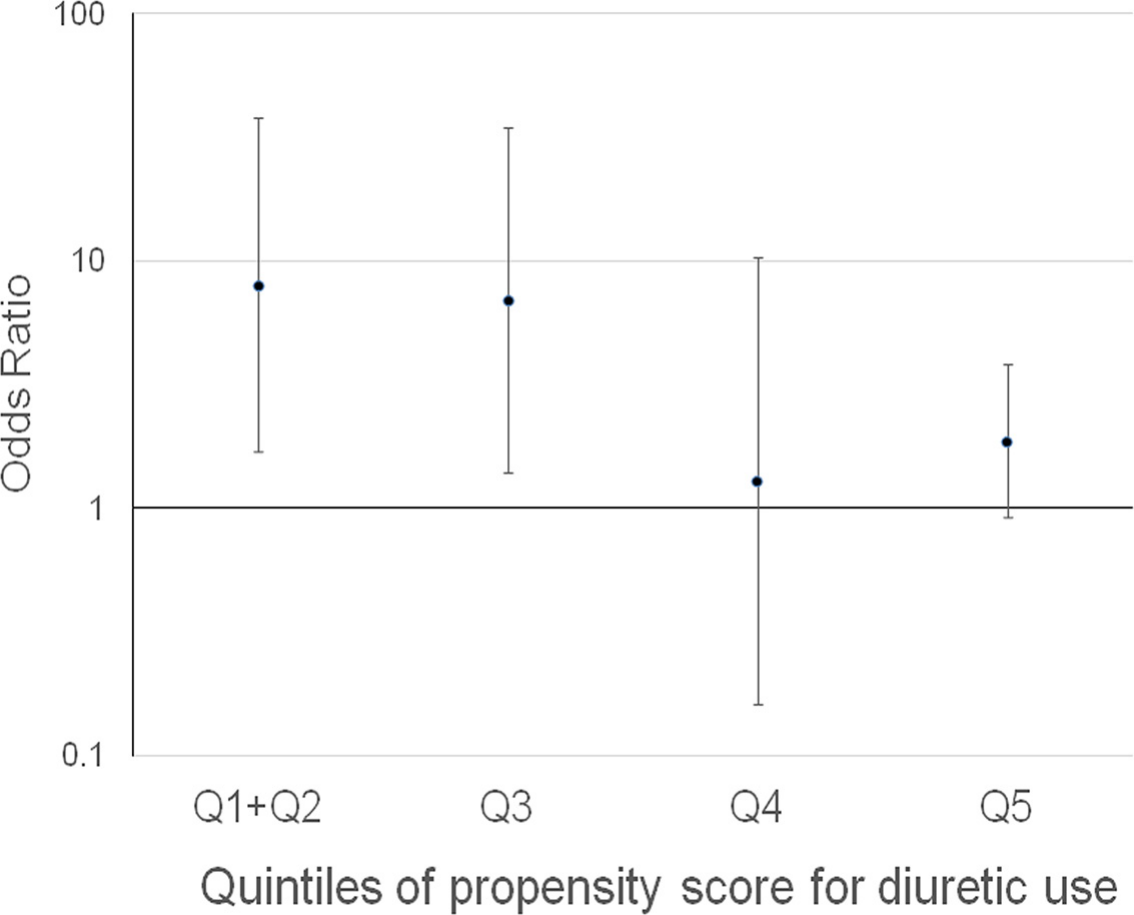
Odds ratio of postoperative acute kidney injury in diuretic user in each quintile of propensity score for diuretic use. p for interaction [PS quintiles * diuretic use] = 0.057<0.1.

To examine whether propensity for diuretic use would affect the effect size of diuretic use, the associations of diuretic use and postoperative AKI were examined in each PS quintile for diuretic use. The use of diuretics was significantly associated with postoperative AKI in the first and second quintiles combined (the first and second quintiles were grouped as the number of outcomes in these quintiles were small) and in the third quintile of PS for diuretic use, but not in other quintiles (Fig 1) (p for interaction [PS quintiles * diuretic use] = 0.057 < 0.1). Thirty-nine patients used ACE-I/ARB and diuretics concomitantly. Pre- and/or intra-operative use of ACE-I/ARB was a significant effect modifier for the association between diuretics use and postoperative AKI (p for interaction [ACE-I/ARB*diuretics] = 0.007). The use of diuretics was significantly associated with postoperative AKI among non-users of ACE-I/ARB but not among users of ACE-I/ARB (Table 4).

**Table 4:** Odds ratio of postoperative acute kidney injury in diuretic users stratified by the use of ACE-I/ARB.

	Odds ratio	95% CI	p
ACE-I/ARB (-)	4.16	2.00–8.64	<0.001
ACE-I/ARB (+)	0.44	0.12–1.61	0.22

ACE-I; angiotensin-converting enzyme inhibitor, ARB: angiotensin receptor blocker

P for interaction (ACE-I/ARB*diuretics) = 0.007

Among 99 diuretic users, 63 patients used loop diuretics, 23 used thiazides, 26 used potassium-sparing diuretics, and one used carperitide (the sum exceeds 99 as 13 received both a loop and a potassium-sparing diuretics and 1 received both a thiazide and a potassium-sparing diuretics). The odds ratios (95% CI) of postoperative AKI among users compared with non-users were 2.64 (1.28–5.43), 1.80 (0.50–6.48), and 1.33 (0.36–4.87) for loop diuretics, thiazides, and potassium-sparing diuretics, respectively.

## Discussion

Diuretics, ACE-I and ARB can possibly increase the risk of postoperative AKI by their hemodynamic effect on kidneys. In this study, pre- and/or intra-operative use of diuretics, but not ACE-I/ARB, was significantly associated with the development of AKI after non-cardiac surgery. Diuretic use was significantly associated with postoperative AKI among patients with low propensity for diuretic use.

Several previous studies addressed whether diuretics, ACE-I or ARB were associated with AKI after cardiac surgery. Preoperative use of diuretics was not a significant predictor of AKI after cardiac surgery [15, 18, 19]. The association of preoperative ACE-I/ARB use and AKI after cardiac surgery has varied among studies, with results showing positive [17, 22], negative [19], and no association [6, 15, 16, 18]. The reasons for this discrepancy are not completely clear. The definitions of AKI were different (requirement of renal replacement therapy, RIFLE criteria or AKI network criteria). Previous studies showed that ACE-I was associated with decreased systemic vascular resistance and increased cardiac index and creatinine clearance after on-pump coronary artery bypass surgery in patients with left ventricular dysfunction (left ventricular ejection fraction < 40%) [35, 36]. Whether ACE-I or ARB increases or decreases the risk of postoperative AKI might be determined by the balance between their beneficial effects on cardiac function and their effects of decreasing glomerular filtration pressure via dilation of efferent arterioles. The surgical techniques used in these studies also differed (on-pump, off-pump or both). Cardiopulmonary bypass was shown to increase plasma renin activity [36] and thus the effects of ACE-I or ARB could differ between on- and off-pump cardiac surgery.

On the other hand, patients undergoing non-cardiac surgery are less likely to have left ventricular dysfunction than those undergoing cardiac surgery. We speculated that the hemodynamic effect of these agents on kidneys would predominate and that they would be independently associated with AKI after non-cardiac surgery. Pre- and/or intra-operative use of diuretics was significantly associated with postoperative AKI, but contrary to our hypothesis, the use of ACE-I/ARB was not. Our cohort included only 7 (0.3%) patients with left ventricular ejection fraction of 40% or less and 396 (14.5%) patients with eGFR less than 60 ml/min/1.73m^2^, both groups at high risk of developing AKI. As a result, the proportion of patients in this study who developed postoperative AKI was much lower (5.0%) than those in prior studies who underwent cardiac surgery (20–40%) [17, 18], despite the use of the same AKI network criteria for the definition of AKI. Patients in our cohort might have had sufficient renal reserve and thus the risk of developing postoperative AKI might not have been increased by the preoperative use of ACE-I/ARB. It is of note, however, that the use of diuretics was significantly associated with the development of postoperative AKI though patients in our study seemed to have renal reserve. During the postoperative period, fluids shift from the intravascular space to the third space and administration of diuretics may exacerbate the intravascular volume contraction. This may explain the significant increase in the risk of postoperative AKI observed in our study.

Our results differed from those of several previous studies focusing on non-cardiac surgery. The preoperative use of ACE-I/ARB was shown to be an independent predictor of postoperative AKI (defined by AKIN network criteria) in elective orthopedic surgery [26], lung resection surgery [28], and esophageal cancer surgery [30]. The sample sizes and number of outcomes (postoperative AKI) were smaller than in our study and logistic regression models were either overfitted [26] or insufficiently adjusted [28]. The study by Lee EH et al [30] was limited by a large percentage of patients with missing data (25–30%) and the use of multiple imputations, and preoperative chemotherapy might have contributed to postoperative AKI. In this study, we performed not only logistic regression analyses but also PS adjustment, PS matching, and inverse probability weighting to confirm the results and there were a very small number of patients with missing data. Shah M et al. showed that preoperative use of ACE-I/ARB was significantly associated with a lower incidence of postoperative AKI (defined by need for renal replacement therapy) [37] after major elective surgery. Their study included both patients undergoing cardiac and non-cardiac surgery and there was no subgroup analysis in the non-cardiac surgery cohort. They also showed that the presence of CKD was a significant effect modifier and that this association between ACE-I/ARB use and postoperative AKI was primarily evident in patients with CKD. In our study, the presence of CKD was not a significant effect modifier.

We found that diuretic use was significantly associated with postoperative AKI but only in patients with a low propensity for diuretic use (Fig 1). The preoperative use of ACE-I/ARB was also a significant effect modifier for the association between diuretic use and postoperative AKI. The use of diuretics was significantly associated with postoperative AKI only among non-users of ACE-I/ARB (Table 4). This is consistent with the fact that users of ACE-I/ARB are more likely to have heart failure, hypertension and CKD, which corresponds to patients with a high propensity for diuretic use. Patients with a high propensity for diuretic use, such as those with congestive heart failure and/or CKD, are at high risk of developing volume overload. It is suggested that elevated venous pressure is associated with worsening renal function, and animal studies demonstrated improvements in renal function after venous pressure was lowered [38–40]. Thus, in patients with high propensity for diuretic use, the benefit of avoiding volume overload might have offset the risk of volume contraction. On the other hand, in patients with a low propensity for diuretic use, such as those with essential hypertension or those who received diuretics only to maintain urine output, diuretics might have resulted in intravascular volume contraction and caused postoperative AKI.

Among the different classes of diuretics, only loop diuretics were significantly associated with postoperative AKI. This is likely because it is the most potent diuretic and it causes greater intravascular volume contraction than other agents [41]. However, it is possible that the observed difference was due to the lack of statistical power with thiazides or other agents, leading to low accuracy of parameter estimates.

The strength of our study is that our cohort included a large number of patients who underwent non-cardiac surgery at a community hospital and who were representatives of those being treated in a general medical practice setting. We selected as many potential predictors of post-operative AKI as possible based on previous studies [5–30], and the covariates used for multivariable logistic regression analyses and PS estimation were more complete than those in previous studies [5–30]. Multivariable logistic regression analyses, PS adjustment, PS matching, and inverse probability weighting yielded similar results. Our study also had several limitations. As this was an observational study, the possibility of unknown confounders cannot be excluded. The low incidence of dialysis requirement and in-hospital mortality precluded the analysis of associations between the use of diuretics or ACE-I/ARB and these outcomes. Also, the relatively small number of patients with CKD precluded subgroup analysis in these patients. PS was estimated using the demographics at the time of preoperative evaluation, not those at the time of drug initiation.

## Conclusion

In our cohort with relatively preserved cardiac and renal function, pre- and/or intra-operative use of diuretics, but not ACE-I/ARB, was significantly associated with the development of AKI following non-cardiac surgery. The effect size of diuretics was greater in patients with low propensity for diuretic use. While the observational nature of our study did not establish causality and randomized controlled trials are warranted, it may be prudent to withhold diuretics preoperatively or to refrain from administering diuretics with the sole goal of maintain urine output in non-cardiac surgery, considering the minimal downsides of this approach in patients with preserved cardiac and renal function.

## Supporting Information

S1 Fig Odds ratio (95% CI) of postoperative acute kidney injury for each quintile of propensity score (Q1 as a reference) for diuretics and ACE-I/ARB. In unadjusted model, only quintiles of PS were included as covariates. In adjusted model, the use of diuretics or ACE-I/ARB was also included as a covariate.
(TIF)

S1 Table Demographics of the patients matched on propensity score for diuretic use. (DOCX)

S2 Table Demographics of the patients matched on propensity score for ACE-I/ARB use. (DOCX)

S3 Table Odds ratio of postoperative acute kidney injury by inverse-probability weight test. (DOCX)
