# Supplementary figures and images for: Pre- and/or Intra-Operative Prescription of Diuretics, but Not Renin-Angiotensin-System Inhibitors, Is Significantly Associated with Acute Kidney Injury after Non-Cardiac Surgery: A Retrospective Cohort Study
Source: PLoS One. 2015 Jul 6;10(7):e0132507. doi: 10.1371/journal.pone.0132507 (PMC4492997; doi:10.1371/journal.pone.0132507)

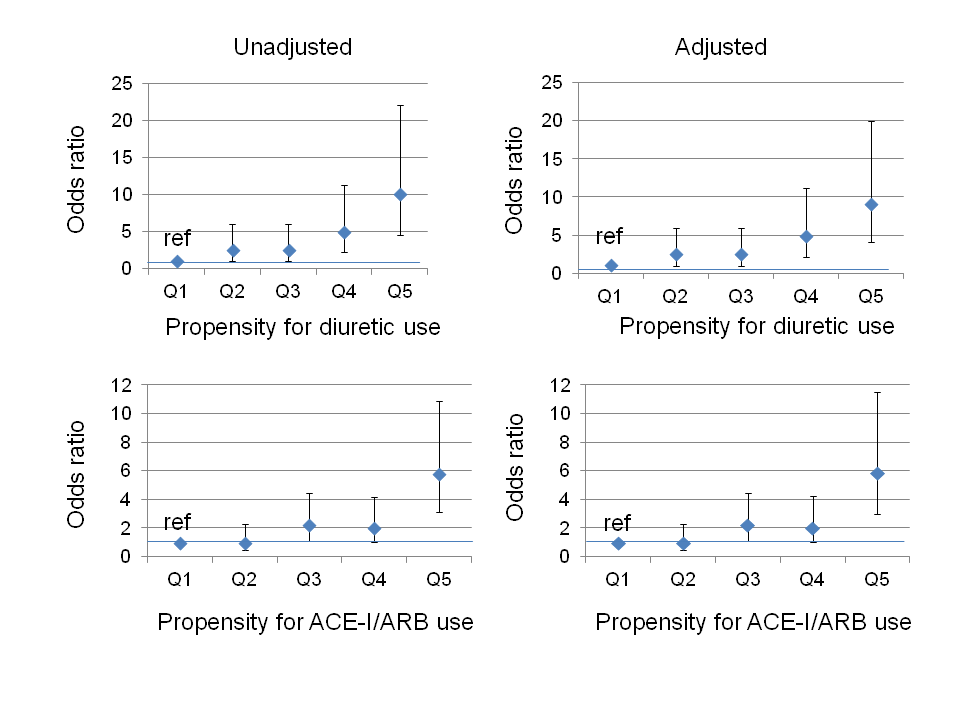

Supplement: S1 Fig — In unadjusted model, only quintiles of PS were included as covariates. In adjusted model, the use of diuretics or ACE-I/ARB was also included as a covariate. (TIF) [file pone.0132507.s001.tif]
