# Supplementary material for: Pre- and/or Intra-Operative Prescription of Diuretics, but Not Renin-Angiotensin-System Inhibitors, Is Significantly Associated with Acute Kidney Injury after Non-Cardiac Surgery: A Retrospective Cohort Study
Source: PLoS One. 2015 Jul 6;10(7):e0132507. doi: 10.1371/journal.pone.0132507 (PMC4492997; doi:10.1371/journal.pone.0132507)
Supplement: S1 Table — (DOCX) [file pone.0132507.s002.docx]

S1Table. Demographics of the patients matched on propensity score for diuretic use

|  | Diuretics  (n=94) | No diuretics  (n=188) | p |
| --- | --- | --- | --- |
| Age | 71.5 (61.8-80.0) | 69.0 (62.0-76.0) | 0.14 |
| Male sex | 53 (56.4) | 112 (59.6) | 0.61 |
| Intra-thoracic surgery  Intra-abdominal surgery  Surgery with large fluid shift  Others | 14 (14.9)  51 (54.3)  12 (12.8)  17 (18.1) | 29 (15.4)  108 (57.4)  21 (11.2)  30 (16.0) | 0.93 |
| Emergent surgery | 8 (8.5) | 11 (5.9) | 0.40 |
| eGFR (ml/min/1.73m^2^) | 72.0 (55.8-87.6) | 70.8 (55.9-84.1) | 0.48 |
| Body mass index | 22.0 (19.0-25.3) | 22.5 (20.7-25.5) | 0.31 |
| Smoking | 20 (21.3) | 45 (23.9) | 0.62 |
| Diabetes Mellitus | 27 (28.7) | 65 (34.6) | 0.32 |
| Insulin | 7 (7.4) | 18 (9.6) | 0.55 |
| Hypertension | 59 (62.8) | 110 (58.5) | 0.49 |
| COPD | 14 (14.9) | 20 (10.6) | 0.30 |
| Atrial fibrillation | 10 (10.6) | 17 (9.0) | 0.67 |
| Peripheral arterial disease | 1 (1.1) | 1 (0.5) | 0.62 |
| Cerebrovascular disease | 10 (10.6) | 10 (5.3) | 0.10 |
| Coronary artery disease | 11 (11.7) | 18 (9.6) | 0.58 |
| Hematocrit (%) | 37.3 (32.0-41.0) | 37.0 (33.0-40.4) | 0.87 |
| INR > 1.5 | 3 (3.2) | 4 (2.1) | 0.59 |
| Platelet < 150,000/μl | 17 (18.1) | 48 (25.5) | 0.16 |
| Vasopressors | 62 (66.0) | 124 (66.0) | 1.00 |
| Left ventricular ejection fraction  >40%  <=40%  missing | 63 (67.0)  1 (1.1)  30 (31.9) | 116 (61.7)  2 (1.1)  70 (37.2) | 0.68 |
| NSAIDs | 78 (83.0) | 152 (80.9) | 0.66 |
| Contrast | 7 (7.4) | 16 (8.5) | 0.76 |
| ACE-I/ARB | 35 (37.2) | 71 (37.8) | 0.93 |

Data are shown as median (interquartile range) or number (%). P values are by Mann-Whitney U test or Chi-square test. AKI: acute kidney injury, eGFR: estimated glomerular filtration rate, COPD: chronic obstructive pulmonary disease, INR: international normalized ratio of prothrombin time, NSAIDs: non-steroidal anti-inflammatory drugs, ACE-I: angiotensin converting enzyme inhibitor, ARB: angiotensin receptor blocker
