# Supplementary material for: Pre- and/or Intra-Operative Prescription of Diuretics, but Not Renin-Angiotensin-System Inhibitors, Is Significantly Associated with Acute Kidney Injury after Non-Cardiac Surgery: A Retrospective Cohort Study
Source: PLoS One. 2015 Jul 6;10(7):e0132507. doi: 10.1371/journal.pone.0132507 (PMC4492997; doi:10.1371/journal.pone.0132507)
Supplement: S2 Table — (DOCX) [file pone.0132507.s003.docx]

S2 Table. Demographics of the patients matched on propensity score for ACE-I/ARB use

|  | ACE-I/ARB  (n=309) | No ACE-I/ARB  (n=618) | p |
| --- | --- | --- | --- |
| Age | 68.0 (62.0-75.0) | 70.0 (61.0-76.0) | 0.66 |
| Male sex | 154 (49.8) | 302 (48.9) | 0.78 |
| Intra-thoracic surgery  Intra-abdominal surgery  Surgery with large fluid shift  Others | 44 (14.2)  146 (47.2)  58 (18.8)  61 (19.7) | 80 (12.9)  306 (49.5)  110 (17.8)  122 (19.7) | 0.90 |
| Emergent surgery | 17 (5.5) | 44 (7.1) | 0.35 |
| eGFR (ml/min/1.73m^2^) | 73.2 (62.0-87.5) | 73.2 (62.3-87.3) | 0.93 |
| Body mass index | 23.4 (21.0-25.6) | 23.4 (21.3-26.2) | 0.61 |
| Smoking | 60 (19.4) | 109 (17.6) | 0.51 |
| Diabetes Mellitus | 71 (23.0) | 136 (22.0) | 0.74 |
| Insulin | 11 (3.6) | 17 (2.8) | 0.50 |
| Hypertension | 295 (95.5) | 595 (96.3) | 0.55 |
| COPD | 33 (10.7) | 66 (10.7) | 1.00 |
| Atrial fibrillation | 10 (3.2) | 25 (4.0) | 0.54 |
| Peripheral arterial disease | 5 (1.6) | 13 (2.1) | 0.61 |
| Cerebrovascular disease | 19 (6.1) | 44 (7.1) | 0.58 |
| Coronary artery disease | 29 (9.4) | 62 (10.0) | 0.76 |
| Hematocrit (%) | 38.2 (35.3-41.6) | 38.8 (35.5-41.9) | 0.38 |
| INR > 1.5 | 2 (0.6) | 6 (1.0) | 0.62 |
| Platelet < 150,000/μl | 31 (10.0) | 68 (11.0) | 0.65 |
| Vasopressors | 178 (57.6) | 350 (56.6) | 0.78 |
| Left ventricular ejection fraction  >40%  <=40%  missing | 206 (66.7)  1 (0.3)  102 (33.0) | 409 (66.2)  0  209 (33.8) | 0.36 |
| NSAIDs | 273 (88.3) | 548 (88.7) | 0.88 |
| Contrast | 17 (5.5) | 32 (5.2) | 0.84 |
| Diuretics | 15 (4.9) | 31 (5.0) | 0.92 |

Data are shown as median (interquartile range) or number (%). P values are by Mann-Whitney U test or Chi-square test. AKI: acute kidney injury, eGFR: estimated glomerular filtration rate, COPD: chronic obstructive pulmonary disease, INR: international normalized ratio of prothrombin time, NSAIDs: non-steroidal anti-inflammatory drugs, ACE-I: angiotensin converting enzyme inhibitor, ARB: angiotensin receptor blocker
