# Supplementary material for: Pre- and/or Intra-Operative Prescription of Diuretics, but Not Renin-Angiotensin-System Inhibitors, Is Significantly Associated with Acute Kidney Injury after Non-Cardiac Surgery: A Retrospective Cohort Study
Source: PLoS One. 2015 Jul 6;10(7):e0132507. doi: 10.1371/journal.pone.0132507 (PMC4492997; doi:10.1371/journal.pone.0132507)
Supplement: S3 Table — (DOCX) [file pone.0132507.s004.docx]

S3 Table. Odds ratio of postoperative acute kidney injury by inverse-probability weight test

|  |  | Odds ratio (95 % CI) | n |
| --- | --- | --- | --- |
| Analysis 1 | ACE-I/ARB  Diuretics | 1.374 (0.898-2.102)  2.498 (1.153-5.408) | 2666  2667 |
| Analysis 2 | ACE-I/ARB  Diuretics | 0.974 (0.519-1.830)  2.925 (1.276-6.701) | 1680  2480 |

Analysis 1: Excluding patients whose inverse-probability weight < 1 percentile or > 99 percentile

Analysis 2: Excluding patients whose propensity scores do not overlap among users and non-users of ACE-I/ARB or diuretics

ACE-I: angiotensin converting enzyme inhibitors, ARB: angiotensin receptor blockers
